# Chelation of hippocampal zinc enhances long‐term potentiation and synaptic tagging/capture in CA1 pyramidal neurons of aged rats: implications to aging and memory

**DOI:** 10.1111/acel.12537

**Published:** 2016-09-16

**Authors:** Mahesh Shivarama Shetty, Mahima Sharma, Sreedharan Sajikumar

**Affiliations:** ^1^ Department of PhysiologyYong Loo Lin School of MedicineNational University of SingaporeBlock MD9, 2 Medical DriveSingapore117 597Singapore; ^2^ Neurobiology/Aging ProgramLife Sciences Institute (LSI)National University of Singapore#04‐44, 28 Medical DriveSingapore117 456Singapore

**Keywords:** aging, D1/D5 receptor, dopamine, long‐term potentiation, synaptic tagging and capture, zinc

## Abstract

Aging is associated with decline in cognitive functions, prominently in the memory consolidation and association capabilities. Hippocampus plays a crucial role in the formation and maintenance of long‐term associative memories, and a significant body of evidence shows that impairments in hippocampal function correlate with aging‐related memory loss. A number of studies have implicated alterations in hippocampal synaptic plasticity, such as long‐term potentiation (LTP), in age‐related cognitive decline although exact mechanisms underlying are not completely clear. Zinc deficiency and the resultant adverse effects on cognition have been well studied. However, the role of excess of zinc in synaptic plasticity, especially in aging, is not addressed well. Here, we have investigated the hippocampal zinc levels and the impairments in synaptic plasticity, such as LTP and synaptic tagging and capture (STC), in the CA1 region of acute hippocampal slices from 82‐ to 84‐week‐old male Wistar rats. We report increased zinc levels in the hippocampus of aged rats and also deficits in the tetani‐induced and dopaminergic agonist‐induced late‐LTP and STC. The observed deficits in synaptic plasticity were restored upon chelation of zinc using a cell‐permeable chelator. These data suggest that functional plasticity and associativity can be successfully established in aged neural networks by chelating zinc with cell‐permeable chelating agents.

## Introduction

Zinc, in addition to its plethora of physiological functions in the brain, is involved in the formation and maintenance of long‐term memories. Zinc is abundant in the brain where most of it (~90%) is found in complexes with proteins, acting as a structural or catalytic cofactor (Huang, 1997; Takeda, 2000). This complexed zinc pool is regarded histochemically invisible. In certain regions of the brain, such as the cerebral cortex and limbic regions, zinc is present in its free, ionic form (Zn^2+^) within the synaptic vesicles (Frederickson & Danscher, 1990; Takeda & Tamano, 2014). This constitutes the chelatable or histochemically reactive zinc pool. These neurons that release Zn^2+^ are mostly glutamatergic and are thus sometimes referred to as ‘glucinergic’ or ‘zincergic’ neurons.

The hippocampus and amygdala are two important brain regions involved in learning and memory formation and are enriched with histochemically reactive zinc (Frederickson & Danscher, 1990). Vesicular zinc can be detected in the trisynaptic circuits of the hippocampus. Zinc is co‐released with glutamate in an activity‐dependent manner from these neuronal terminals (Ueno *et al*., 2002; Takeda & Tamano, 2014). Once released, it is quickly taken up into presynaptic and postsynaptic neurons and astrocytes (Takeda & Tamano, 2014). In addition, zinc modulates the activities of several important receptors, including the α‐amino‐3‐hydroxy‐5‐methyl‐4‐isoxazolepropionic acid/kainate receptors, *N*‐methyl‐d‐aspartate (NMDA) receptors, and γ‐amino butyric acid (GABA_A_) receptors in the extracellular compartment (Smart *et al*., 1994). Zinc not only is released as a neurotransmitter, but also functions as a secondary messenger in neurons and has a functional role in synaptic plasticity and memory (Takeda & Tamano, 2014). Zinc homeostasis is critical for brain function and is tightly regulated by the blood–brain barrier and a host of transporters and zinc‐binding proteins (Takeda, 2000; Takeda *et al*., 2001). Alterations in zinc homeostasis may lead to adverse complex implications and neurological diseases (Barnham & Bush, 2008; Takeda & Tamano, 2014).

The hippocampus primarily appears to be the most responsive region to either a deficiency or an excess of zinc (Suh *et al*., 2009; Takeda & Tamano, 2014). Perturbation toward zinc homeostasis in hippocampal neurons is also linked to cognitive decline under stress or in pathological conditions. For instance, it has been reported that excess intracellular zinc signaling induced by corticosterone and/or stress results in the impairment of hippocampal long‐term potentiation (LTP), an important cellular correlate of learning and memory (Takeda & Tamano, 2014). Interestingly, rats exhibiting elevated levels of dietary zinc show high levels of zinc in the brain and show cognitive and memory deficits (Flinn *et al*., 2005). A zinc‐enriched diet leads to spatial memory deficits in wild‐type mice and potentiates the deficits in transgenic Alzheimer's disease (AD) models (Linkous *et al*., 2009). Excess intracellular Zn^2+^ signaling also leads to a negative cross talk that suppresses Ca^2+^ signaling, which then impairs synaptic plasticity (Takeda & Tamano, 2014).

Aging is associated with structural and functional changes in the brain that could be related to zinc homeostasis (Brown & Dyck 2003; Sensi *et al*., 2011). It has been proposed that brain zinc levels may rise with age possibly leading to age‐related memory loss and neurodegenerative diseases (Sensi *et al*., 2011). A compromised blood–brain barrier, reduced levels or activity of transporters, amyloid deposition, and oxidative stress may all contribute to dyshomeostasis. Alzheimer's disease is an exemplar of a condition of age‐associated cognitive impairment and increased zinc levels are observed in the hippocampus and amygdala of AD patients (Thompson *et al*., 1988; Danscher *et al*., 1997). Furthermore, extracellular Zn^2+^ can be sequestered by amyloid oligomers and thus can precipitate plaque formation (Bush *et al*., 1994; Deshpande *et al*., 2009). Zinc supplementation causes spatial memory deficits in a model of late‐onset AD, which correlates with levels of amyloid‐beta (Flinn *et al*., 2014).

The widely studied mouse models of accelerated aging, senescence‐accelerated mouse prone‐8 and 10 (SAMP8 and SAMP10), show different aspects of brain aging and zinc homeostasis (Saito *et al*., 2000; Onozuka *et al*., 2002). Particularly, Zn^2+^ modulates NMDAR function (Paoletti *et al*., 1997; Izumi *et al*., 2006; Zhu *et al*., 2012) and the subunit composition of NMDARs changes with aging to a predominantly GluN2A/GluN2B composition. GluN2A‐containing NMDARs are much more sensitive to modulation by Zn^2+^. However, it is not clear whether alterations in synaptic plasticity and related mechanisms in advanced aging are associated with high zinc levels. We hypothesized that if indeed hippocampal zinc levels are increased in aging then chelation of excess zinc may restore plasticity deficits in aged neural networks. Thus, we investigated synaptic plasticity mechanisms such as protein synthesis‐dependent late‐LTP (L‐LTP), dopaminergic receptor‐dependent LTP (DA‐LTP), and its associative plasticity mechanisms such as synaptic tagging and capture (STC). STC model (Frey & Morris, 1997, 1998a; Barco *et al*., 2002, 2008) proposes that glutamatergic activation during LTP induction or memory encoding results in instantaneous local ‘tagging’ of activated synapses. These ‘synaptic tags’ later ‘capture’ the diffusely transported plasticity‐related products (PRPs; mRNA or proteins) synthesized in soma or local dendritic domains (for reviews, see Redondo & Morris (2011) and Sajikumar (2015)). STC provides a conceptual basis for how short‐term plasticity/memory transforms to long‐term‐plasticity/memory in a time‐dependent manner and is considered as an important cellular correlate of associative plasticity/memory formation (Redondo & Morris, 2011). Using electrically induced LTP, DA‐LTP, and STC as models, we investigated the beneficial aspects of zinc chelation in aging and our findings indicate that regulating zinc levels in an aging hippocampus with zinc chelators can re‐establish plasticity and associativity, which are impaired in 82‐ to 84‐week‐old rats.

## Results

### High‐frequency stimulation (HFS)‐induced LTP and D1/D5 receptor agonist‐induced LTP are impaired in aged rats

**Figure 1:**
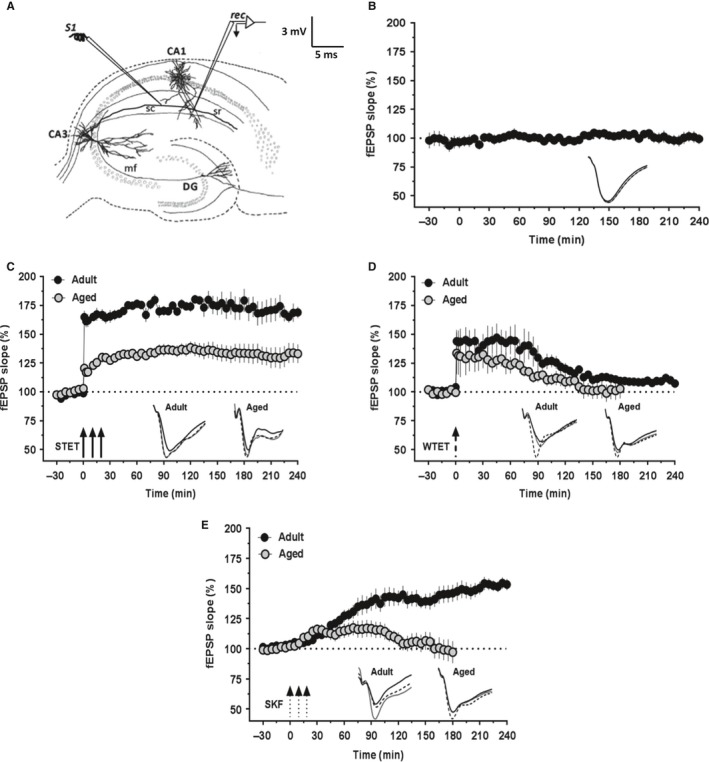
High‐frequency stimulation (HFS)‐induced LTP and D1/D5 receptor agonist‐induced (DA‐LTP) potentiation in hippocampus are impaired in aged rats. (A) Schematic representation of a hippocampal slice showing the location of electrodes in CA1 region for field‐EPSP recording: stimulating electrode (S1) was positioned in stratum radiatum to stimulate Schaffer collaterals, and recording electrode (rec) was positioned onto CA1 apical dendrites. (B) Stable long‐term baseline recordings from the slices of aged rats (*n* = 10). The test stimulation was given every 5 min. The slope values did not show statistically significant deviation from the baseline values at any time point (Wilcoxon's test, *P *>* *0.05). (C) L‐LTP induced by a strong tetanization protocol (STET; three arrows represent three HFS trains) in slices from 5‐ to 7‐week‐old rats (filled circles; *n* = 6) and 82‐ to 84‐week‐old rats (open circles; *n* = 9). The magnitude of potentiation was significantly less in the aged rat group compared with the adult rat group (Mann–Whitney *U*‐test, *P *<* *0.05). (D) E‐LTP induced by a weak tetanization protocol (WTET; broken arrow represents the single weak stimulation train) in slices from adult rats (filled circles; *n* = 6) and aged rats (open circles; *n* = 7). The potentiation was slightly lesser in the slices of aged rats compared with that in adult group and decayed to baseline comparatively quickly, by 100 min (Wilcoxon's test, *P *>* *0.05), whereas in adult slices it decayed to baseline after 150 min (Wilcoxon's test, *P *>* *0.05). (E) Slow‐onset potentiation induced by bath application of the dopamine D1/D5 receptor agonist SKF‐38393 (SKF; three broken arrows represent the spaced stimulation) in slices from adult rats (filled circles; *n* = 8) and aged rats (open circles; *n* = 8). The potentiation was only transient in the slices of aged rats and decayed to the baseline quickly by 100 min, whereas it lasted over 4 h in the slices of aged rats. Error bars in all the graphs indicate ±SEM. Insets show representative fEPSP traces recorded at baseline (black solid line), 30 min (dotted line), and at the end of recording period (gray solid line). Scale bar for all the traces: 3 mV/5 ms.

In the first series of experiments, we investigated the HFS‐induced LTP [both early‐LTP (E‐LTP) and L‐LTP] and the slow‐onset potentiation induced by the bath application of D1/D5 receptor agonist (DA‐LTP) in the CA1 Schaffer collateral (SC) synapses of the 82‐ to 84‐week‐old (aged) and 5‐ to 7‐week‐old (young adult) rats. As a control experiment, long‐term baseline recording was performed in the slices of aged rats and the recordings were stable over 4 h (Fig. 1B; *n* = 10; Wilcoxon's test, *P *>* *0.05). In adult rats, three spaced trains of HFS (STET) resulted in long‐lasting L‐LTP (Fig. 1C, filled circles; *n* = 6). The mean field‐EPSP (fEPSP) slope value immediately after first tetanization was 164.75 ± 5.5% (Wilcoxon's test, *P *=* *0.018) and it remained at significantly different levels compared with the baseline, over 4 h (168.83 ± 4.8%, Wilcoxon's test, *P *=* *0.018). The same paradigm of stimulation also resulted in L‐LTP in the slices of aged rats (Fig. 1C, open circles; *n* = 9). However, the magnitude of potentiation was less in the aged rat slices compared with that in the slices of adult rats (Mann–Whitney *U*‐test; *P *<* *0.05), which is consistent with our earlier report (Sharma *et al*., 2015). The mean potentiation immediately after the first tetanization was 120.38 ± 3.6% (Wilcoxon's test, *P *=* *0.004) and the potentiation remained at significant levels, compared with the baseline values, for the entire recording period (4 h; 133.02 ± 8.1%, Wilcoxon's test, *P *=* *0.004).

Next, we induced E‐LTP in the slices of aged and adult rats using a weak tetanization (WTET) protocol. In the slices of adult rats, WTET resulted in significant potentiation (Fig. 1D, filled circles; +1 min: 143.96 ± 9.7%, Wilcoxon's test, *P *=* *0.027; *n* = 6), which decayed to the baseline gradually after 150 min. The potentiation at the end of 4 h was not significantly different from the baseline (107.23 ± 1.9%, Wilcoxon's test, *P *=* *0.138). In the slices of aged rats also, WTET resulted in a transient E‐LTP (Fig. 1D, open circles; +1 min: 133.56 ± 18.1%, Wilcoxon's test, *P *=* *0.031, *n* = 7). The potentiation was slightly lesser compared with that in adult group and decayed to baseline comparatively quickly by 100 min (Wilcoxon's test, *P *>* *0.05). At the end of 3 h, the mean fEPSP slope values remained at the baseline values (102.74 ± 8.3%; Wilcoxon's test, *P *=* *0.375).

It has been reported earlier that dopamine or its agonists induce slow‐onset potentiation that shares mechanisms common to that of tetani‐induced LTP (Huang & Kandel, 1995). We tested the duration and maintenance of DA‐LTP in the CA1 SC synapses. After a 30‐min stable baseline recording, dopamine D1/D5‐receptor agonist SKF‐38393 (SKF; 50 μm) was bath‐applied for three 5‐min durations with an interval of 5 min between applications, a paradigm similar to the three repeated HFS protocol used for the induction of conventional L‐LTP (Navakkode *et al*., 2007, 2012; Shivarama Shetty *et al*., 2016). In the slices of adult rats, bath application of SKF leads to a slow‐onset potentiation, the time course of which was similar to that observed in our earlier report (Shivarama Shetty *et al*., 2016). The potentiation became statistically significant by 25 min (Fig. 1E, filled circles; 111.59 ± 2.4%, Wilcoxon's test, *P *=* *0.049, *n* = 8) and maintained over 4 h (153.20 ± 4.5%, Wilcoxon's test, *P *=* *0.011). In the slices of aged rats, however, the potentiation induced was significantly different from the baseline values rather slowly by 65 min (Fig. 1E, open circles; 115.75 ± 5.71%, Wilcoxon's test, *P *=* *0.035, *n* = 8) and only up to 100 min (115.54 ± 6.1%, Wilcoxon's test, *P *=* *0.035). Thus, the potentiation was only transient and the mean fEPSP slope value was not significantly different from the baseline at the end of 3 h (97.07 ± 9.1%, Wilcoxon's test, *P *=* *0.945).

### Associative properties of HFS‐induced and D1/D5 receptor‐mediated potentiation are deficient in the CA1 region of aged rats

**Figure 2:**
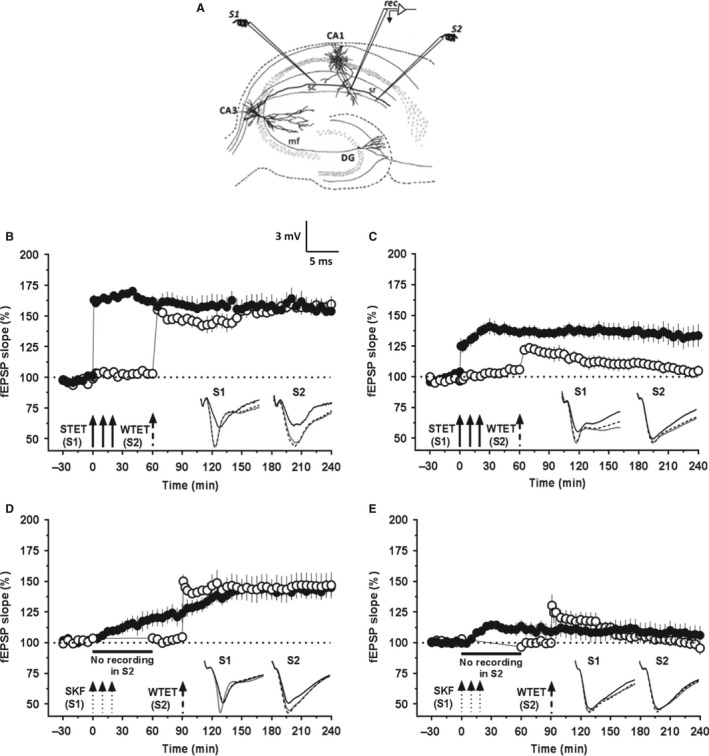
Synaptic tagging and capture by HFS‐induced L‐LTP and DA‐LTP is impaired in hippocampal CA1 region of aged rats. (A) Schematic representation of a hippocampal slice depicting the location of electrodes in CA1 region for the synaptic tagging and capture (STC) experiments with two inputs. S1 and S2 are two stimulating electrodes positioned in the stratum radiatum to stimulate two independent synaptic inputs to the same neuronal population. The recording electrode is placed midway between S1 and S2 to record fEPSP from the apical dendrites. In (B) and (C), after a stable baseline recording of 30 min in S1 and S2, L‐LTP was induced in S1 by STET and 60 min later, WTET was applied to S2 for inducing E‐LTP in this pathway. In (D) and (E), the test stimulation in S2 was silenced just before SKF application for one hour. After resumption and 30 min of baseline recording, E‐LTP was induced in S2. (B) ‘Strong‐before‐weak (SBW)’ stimulation paradigm demonstrating STC in the adult rats (*n* = 6). STET in S1 (filled circles) resulted in a significant potentiation that remained till the end of recording (4 h, Wilcoxon's test, *P *=* *0.027) and also led to the reinforcement of the WTET‐induced E‐LTP in S2 (open circles; 4 h, Wilcoxon's test, *P *=* *0.027). (C) SBW paradigm in aged slices showed impaired STC (*n* = 8). The STET in S1 (filled circles) resulted in L‐LTP that remained over 4 h (Wilcoxon's test, *P *=* *0.016) but did not lead to the reinforcement of the WTET‐induced E‐LTP in S2 (open circles). The potentiation in S2 was significant only until 145 min (Wilcoxon's test, *P *=* *0.039) after which it gradually decayed to baseline (Wilcoxon's test, *P *>* *0.05). (D) In the adult rat slices (*n* = 6), SKF induced a slow‐onset, persistent potentiation in S1 lasting 4 h (filled circles; Wilcoxon's test, *P *=* *0.043) and also transformed the E‐LTP induced in S2 (open circles) into a persistent one (Wilcoxon's test, *P *=* *0.043). (E) In the aged rat slices (*n* = 12), SKF induced a transient potentiation in S1 that decayed to baseline by 4 h (filled circles; Wilcoxon's test, *P *=* *0.381) and failed to reinforce the E‐LTP induced in S2 (open circles, 4 h, Wilcoxon's test, *P *=* *0.851). Insets show representative fEPSP traces for each input recorded at baseline (black solid line), 30 min after the respective tetanization/stimulation in each input (dotted line), and at 240 min (gray solid line). Symbols as in Fig. 1. Scale bar for the traces 3 mV per 5 ms.

Synaptic tagging and capture (STC) experiments provide cellular evidence for how synapse specificity is achieved during a protein synthesis‐independent situation (Frey & Morris, 1997). STC can be studied in the hippocampal networks using weak‐before‐strong (WBS) (Frey & Morris, 1998b) or strong‐before‐weak (SBW) experimental designs. For studying STC using WBS or SBW, a two‐pathway experimental design is widely used. In this design, two stimulating electrodes are used to stimulate two independent synaptic inputs (S1 and S2) that converge to the same neuronal populations (Fig. 2A). These experimental designs enable us to deliver appropriate stimulations to induce E‐LTP and L‐LTP in S1 and S2 at different time intervals to study STC. In this study, we used a SBW paradigm to investigate STC interactions.

First, we studied STC in hippocampal slices from adult rats. After a stable baseline of 30 min in S1 and S2, L‐LTP was induced in S1 by STET (Fig. 2B, filled circles) and 60 min later, a single weak tetanus (WTET) was applied to S2 for inducing E‐LTP in this pathway (Fig. 2B, open circles). The post‐tetanization potential in S1 was statistically significant from immediately after tetanization (162.84 ± 4.51%, Wilcoxon's test, *P *=* *0.015) throughout until the end of recording (153.70 ± 9.14%, Wilcoxon's test, *P *=* *0.015). In S2 also, WTET led to a statistically significant potentiation immediately (155.08 ± 4.82%, Wilcoxon's test, *P *=* *0.015) and it maintained up to the end of the recording period (159.42 ± 6.82%, Wilcoxon's test; *P *=* *0.015). Thus, this procedure resulted in the reinforcement of the E‐LTP in S2 demonstrating STC (Fig. 2B; *n* = 6).

However, the same experimental procedure did not result in STC in the neuronal population of aged rats (Fig. 2C; *n* = 8). The STET‐induced L‐LTP in S1 (filled circles) was intact and was significantly potentiated following tetanization (+1 min; 125.1 ± 6.1%, Wilcoxon's test, *P *=* *0.008) and remained over 4 h (133.5 ± 9%, Wilcoxon's test, *P *=* *0.016). The post‐tetanization potentials in S2 (open circles) were significantly potentiated after the WTET (121.91 ± 3.3%, Wilcoxon's test; *P *=* *0.008) but only until 145 min (111.7 ± 5.2%, Wilcoxon's test, *P *=* *0.039) following which the potentiation decayed rapidly and was at the baseline values at the end of recording (104.83 ± 4.5%, Wilcoxon's test; *P *=* *0.312). We have also reported the failure in expression of STC in the hippocampus of aged rats using a WBS design of STC (Sharma *et al*., 2015).

We then investigated the STC processes initiated by DA‐LTP. Figure 2D represents the experiment to study STC processes initiated by DA‐LTP in hippocampal slices. After recording a stable baseline of 30 min, SKF was applied in three pulses (similar to as in Fig. 1E). While in input S1, potentials were recorded normally (filled circles), test stimulation was suspended in input S2 for 1 h from the beginning of SKF application (Fig. 2D). It has been reported earlier that suspending the test stimulation during the induction of DA‐LTP can prevent the synergistic interactions of NMDAR and D1/D5 receptor function necessary for the induction of the late event resulting in no slow‐onset potentiation in the corresponding input (Navakkode *et al*., 2007, 2012; Shivarama Shetty *et al*., 2016). Test stimulations were resumed in S2 from 60 min onwards, and a baseline of 30 min was recorded before the induction of E‐LTP in S2 by WTET at 90th min (Fig. 2D, open circles). These control experiments performed on adult rat slices showed normal STC (Fig. 2D; *n* = 6), wherein the DA‐LTP in S1 became significant after 30 min and lasted for 4 h (144.44 ± 6.4%, Wilcoxon's test; *P *=* *0.043) and the WTET‐induced E‐LTP in S2 was reinforced to L‐LTP (4 h, 146.73 ± 10.64%, Wilcoxon's test; *P *=* *0.043). Similar experiments were performed in the slices of aged rats (Fig. 2E; *n* = 12). Here, DA‐LTP in the input S1 (filled circles) was transient and the potentiation was statistically significant only between 15 min (108.2 ± 2.7%, Wilcoxon's test; *P *=* *0.034) and 55 min (110.7 ± 5.4%, Wilcoxon's test; *P *=* *0.064). WTET applied to S2 (open circles) at 90th min resulted in E‐LTP which decayed to baseline by 140th min (111.97 ± 4.9%, Wilcoxon's test; *P *=* *0.091). This demonstrated the lack of DA‐LTP‐mediated STC in aged rats.

Together, these results showed that the associative properties such as synaptic tagging and capture are impaired in the aged hippocampal CA1 pyramidal neurons.

### Zn^2+^ levels are increased in the hippocampus of aged rats

**Figure 3:**
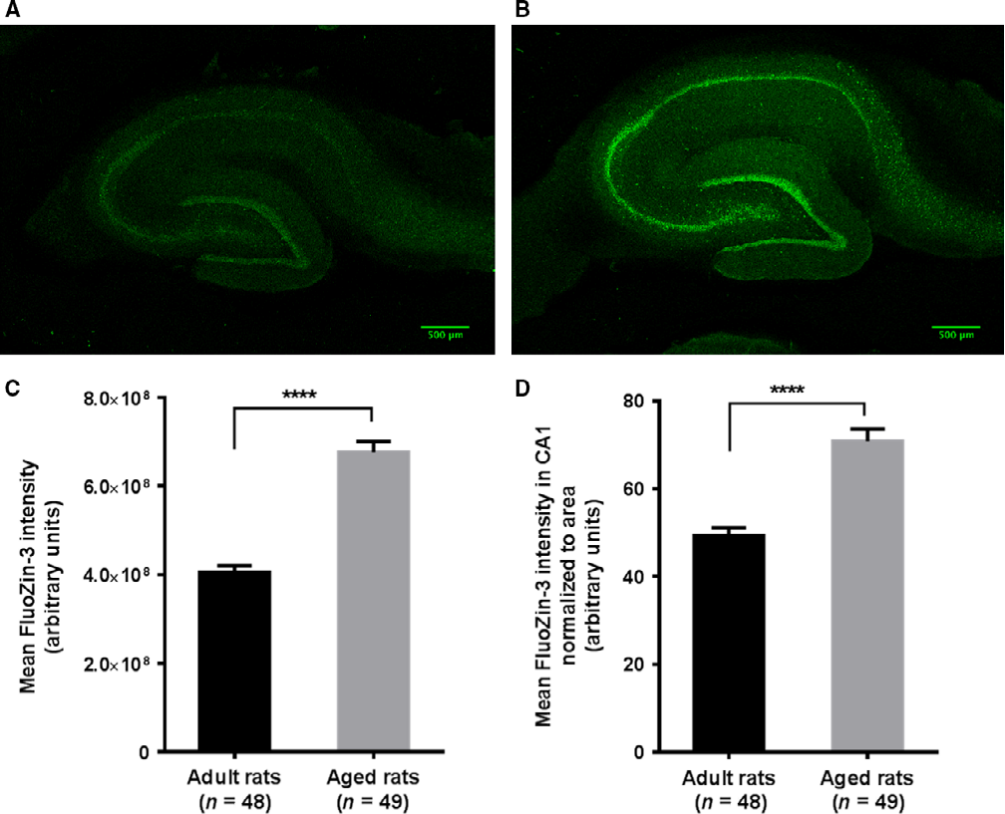
Zn^2+^ levels are increased in the hippocampus of aged rats compared with adult rats. A cell‐permeable fluorescent Zn^2+^ indicator FluoZin‐3 (AM) was used to visualize Zn^2+^ in hippocampus. The acute hippocampal slices (400 μm thick) were incubated in ACSF at 32 °C for 2 h and then stored in 4% paraformaldehyde/PBS at 4 °C. Following three washes in ACSF, slices were treated with 2 μm FluoZin‐3 solution for 10 min in dark, rinsed once with ACSF and imaged with a confocal microscope. (A) FluoZin‐3 fluorescence from a representative hippocampal slice of 5‐ to 7‐week‐old adult rat. (B) FluoZin‐3 fluorescence from a representative hippocampal slice of aged rat (82–84 weeks old). (C) A plot of mean fluorescence intensities (raw intensity density) from slices of adult rats (*n* = 48 slices) and aged rats (*n* = 49 slices). The analysis showed a significant increase in the mean fluorescence intensity in the aged rat group compared with the adult rat group (Unpaired *t*‐test with Welch's correction, *t* = 9.60; ****P <* *0.0001). (D) A plot of mean fluorescence intensities from the CA1 region (raw intensity density normalized to area) from slices of adult and aged rats. A significant increase was observed in the mean fluorescence intensity in the aged rat group compared with the adult rat group (Unpaired *t*‐test with Welch's correction, *t* = 6.44; ****P <* *0.0001).

To investigate our hypothesis that aging is associated with increased hippocampal zinc, we used a cell‐permeable fluorescent indicator of Zn^2+^, FluoZin‐3 (AM) to visualize Zn^2+^ in the hippocampal slices of adult and aged rats. The acute slices, following 2‐h incubation, were preserved in 4% paraformaldehyde (PFA)/PBS at 4 °C until imaging. For imaging, following three washes in artificial cerebrospinal fluid (ACSF), slices were treated with 2 μm FluoZin‐3 solution for 10 min in dark, rinsed once with ACSF, and imaged with a confocal microscope. The slices from the aged rats showed strikingly higher FluoZin‐3 signal than that of adult rats; representative images from adult and aged rats are shown in Fig. 3A,B. The analysis of fluorescence intensity of the whole slice showed a significantly higher mean fluorescence intensity in the aged rat group (*n* = 49 slices from 10 rats) compared with the adult rat group (*n* = 48 slices from nine rats) (Fig. 3C, Unpaired *t*‐test with Welch's correction, *t* = 9.60; ****P <* *0.0001). We also compared the mean fluorescence intensities of FluoZin‐3 signal from the CA1 region of these slices (raw intensity density normalized to area) as our particular interest was in the plasticity of the CA1 pyramidal neurons. This analysis also showed a significant increase in the mean fluorescence intensity in the aged rats compared with the adult rats (Fig. 3D, Unpaired *t*‐test with Welch's correction, *t* = 6.44; ****P <* *0.0001).

These experiments demonstrated increased Zn^2+^ levels in the hippocampus of aged rats, thus supporting our hypothesis that excess zinc could underlie the plasticity impairments in aging.

### Zn^2+^ chelation rescues the deficits in HFS‐LTP and DA‐LTP in aged hippocampal slices

**Figure 4:**
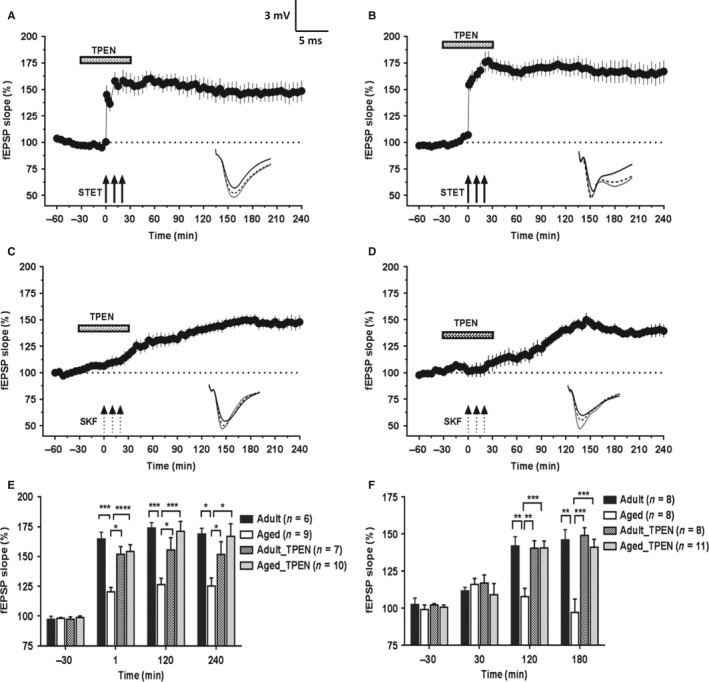
Hippocampal Zn^2+^ chelation restores the deficits in HFS‐induced L‐LTP and DA‐LTP in aged rats. Following 30 min of stable baseline recording, a cell‐permeable Zn^2+^ chelator TPEN (5 μm) was bath‐applied to the slices for a total duration of 1 h. The time of TPEN application is shown with a horizontal bar in each graph. (A) STET‐induced L‐LTP in adult slices in the presence of TPEN (*n* = 7) was not significantly different from the control LTP (Mann–Whitney *U*‐test, *P *>* *0.05). (B) In slices of aged rats, treatment with the Zn^2+^‐chelator TPEN resulted in substantial augmentation of STET‐induced LTP (*n* = 10). (C) Bath application of Zn^2+^‐chelator TPEN along with SKF to slices from adult rats did not affect the potentiation induced. The potentiation followed the same time course as of control DA‐LTP (4 h, Wilcoxon's test, *P *=* *0.011, *n* = 8). (D) Application of SKF to aged slices in the presence of Zn^2+^‐chelator TPEN resulted in restoration of potentiation that lasted for 4 h (*n* = 8); potentiation at the end of 4 h was significantly different from the baseline values (Wilcoxon's test, *P = *0.011). (E) A histogram comparing the mean potentiation at various time points (−30 min, +1 min, +120 min, and +240 min) between STET‐induced L‐LTP in adult and aged slices with or without TPEN treatment. (F) A histogram comparing the mean potentiation at various time points (−30 min, +30 min, +120 min, and +180 min) between SKF‐induced potentiation in adult and aged slices with or without TPEN treatment. Asterisks indicate significant difference between two groups (Mann–Whitney *U*‐test; *P <* *0.05; **P <* *0.01; ***P <* *0.001). Error bars indicate ±SEM. Insets show representative fEPSP traces recorded at baseline (black solid line), 30 minutes after the respective tetanization/stimulation (dotted line), and at 240 min (gray solid line). Symbols as in Fig. 1. Scale bar for the traces 3 mV per 5 ms.

Having observed increased Zn^2+^ levels in the hippocampus of aged rats, next we investigated whether treatment with a Zn^2+^ chelator would have any beneficial effects on the plasticity impairments. We used a cell‐permeable, high‐affinity Zn^2+^ chelator, TPEN. This chelator at a concentration of 5 μm was used in an earlier study to chelate Zn^2+^ in hippocampal mossy fiber synapses (Matias *et al*., 2006). Concentrations ranging from 5 to 25 μm have been used to chelate zinc in previous reports (Xiong *et al*., 1999; Izumi *et al*., 2006). We chose 5 μm concentration and, in the initial series of experiments, checked whether treatment with 5 μm TPEN before and during the STET has any effect on the induced L‐LTP in adult rats (Fig. 4A). After a stable baseline recording of 30 min, TPEN (5 μm) was bath‐applied 30 min before the induction of L‐LTP and was washed out 30 min after the induction of LTP. Thus, the total duration of the treatment with the chelator was 1 h. Applying STET along with TPEN treatment in adult rat slices resulted in normal L‐LTP (Fig. 4A; *n* = 7); the potentials immediately after first tetanization (145.1 ± 8.8%, Wilcoxon's test; *P *=* *0.018) and at the end of 4 h (148.5 ± 9.8%, Wilcoxon's test; *P *=* *0.018) were statistically significant compared with the baseline values. There was no statistically significant difference in the induction and maintenance of Zn^2+^‐chelated L‐LTP (Fig. 4A) compared with the control L‐LTP (Fig. 1C, filled circles) (Mann–Whitney *U*‐test, +1 min *P *=* *0.174; +240 min *P *=* *0.094). We also did not observe any significant changes in the baseline fEPSP slopes during the initial 30‐min treatment with the chelator, before the induction of LTP.

In the next critical series of experiments, we tested the effect of Zn^2+^ chelation on the STET‐induced LTP in the slices from aged rats. The experimental design was the same as of Fig. 4A. Interestingly, the magnitude of STET‐induced LTP in the Zn^2+^ chelator‐treated slices of aged rats (Fig. 4B; *n* = 10) was similar to that of control L‐LTP in adult rat slices (Fig. 1C, filled circles). The potentiation was statistically significant after first tetanization (154.26 ± 5.6%, Wilcoxon's test; *P *=* *0.002) and remained over 4 h (166.89 ± 10.5%, Wilcoxon's test; *P *=* *0.002). There was statistically significant difference in the induction and maintenance of Zn^2+^‐chelated L‐LTP (Fig. 4B) compared with the control L‐LTP (Fig. 1C, open circles) for the entire period of recording (Mann–Whitney *U*‐test, *P *<* *0.05). A histogram comparing the potentiation at various time points (30 min before and 1 min, 2 h, and 4 h after LTP induction) between L‐LTP in adult and aged slices with or without TPEN treatment is shown in Fig. 4E. Five micromolar TPEN alone, applied to the aged rat slices for the same duration of 1 h, showed no significant effects on the baseline synaptic responses (Fig. S1; *n* = 8).

Similar to the experiments with HFS‐induced LTP, we first checked whether chelation of Zn^2+^ with TPEN (5 μm) before and during the induction of DA‐LTP by SKF has any effect on the long‐lasting potentiation induced in the slices of adult rats. Following a stable baseline recording of 30 min, bath application of TPEN was started. After 30 min of TPEN treatment, 50 μm SKF was co‐applied (three 5‐min pulses spaced at 5 min) and the TPEN application was continued until the total duration of the treatment was 1 h (Fig. 4C; *n* = 8). The potentiation followed the usual time course becoming statistically significant by 30 min (116.90 ± 5.5%, Wilcoxon's test, *P *=* *0.046) and remained over the 4 h recorded (147.88 ± 6.7%, Wilcoxon's test, *P *=* *0.011). The treatment with Zn^2+^ chelator did not affect the LTP induced by SKF application in adult slices as there was no significant difference between the potentiation observed with or without Zn^2+^ chelator application [Fig. 1E (filled circles) and Fig. 4C; Mann–Whitney *U*‐test, 4 h, *P *=* *0.862].

Interestingly, SKF application under Zn^2+^‐chelated conditions resulted in a long‐lasting potentiation in the slices of aged rats (Fig. 4D; *n* = 11). Statistically significant potentiation was observed from 80 min (120.84 ± 7.5%, Wilcoxon's test; *P *=* *0.042) until the end of the recording period (139.40 ± 6.1%, Wilcoxon's test, *P *=* *0.001). When compared between the SKF potentiation in aged rat slices with or without TPEN treatment [Fig. 1E (open circles) and Fig. 4D], the potentiation became significantly different 105 min onwards (Mann–Whitney *U*‐test; *P *=* *0.041). A comparison of SKF‐induced potentiation at various time points (30 min before and 30 min, 2 h, and 3 h after SKF application) between adult and aged slices with or without TPEN treatment is shown in Fig. 4F.

### Augmented L‐LTP and re‐established DA‐LTP following Zn^2+^ chelation in slices of aged rats depend on NMDAR activity and protein synthesis

**Figure 5:**
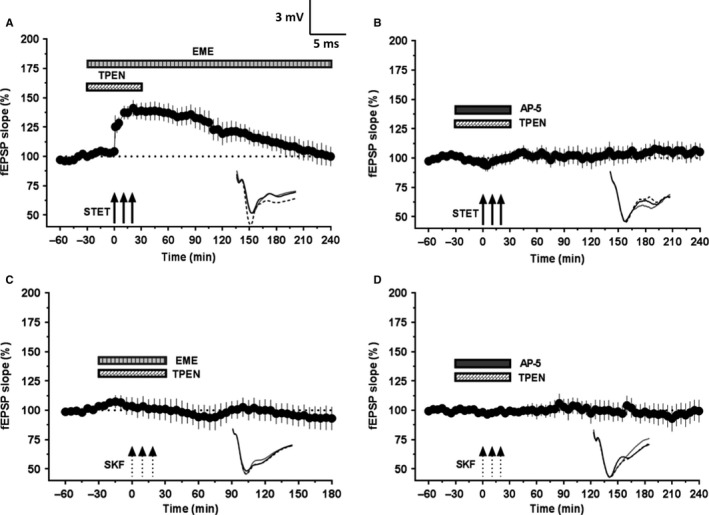
Augmentation of L‐LTP and restoration of DA‐LTP in aged rat slices following Zn^2+^ chelation depends on NMDAR activity and protein synthesis. (A) STET coupled with co‐application of TPEN (5 μm) and emetine (EME, 20 μm) resulted in a potentiation that remained significant for 145 min (*n* = 12; *P *=* *0.042) and decayed to baseline afterward. (B) STET failed to induce potentiation in the presence of NMDAR antagonist AP5 (50 μm) and TPEN (5 μm). The fEPSP response throughout the recording period was not significantly different from that of baseline (*P *>* *0.05, *n* = 8). (C) SKF application coupled with application of TPEN (5 μm) and emetine (20 μm) completely suppressed the DA‐LTP (Wilcoxon's test, *n* = 6; *P *>* *0.05). (D) SKF application failed to induce potentiation in the presence of NMDAR antagonist AP5 (50 μm) and TPEN (5 μm). The fEPSP response at the end of 4 h was not significantly different from that of baseline values (Wilcoxon's test, *P *>* *0.05, *n* = 9). Error bars in all graphs represent ±SEM. Insets show representative fEPSP traces recorded at baseline (black solid line), 30 min after the respective tetanization/stimulation (dotted line), and at 240 min (gray solid line). Symbols as in Fig. 1. Scale bar for the traces 3 mV per 5 ms.

Next, we addressed the question of whether the augmented L‐LTP observed in Zn^2+^ chelator‐treated slices of aged rats required NMDAR activity for its induction and protein synthesis for its persistence. As shown in Fig. 5A, bath application of a protein synthesis inhibitor emetine (20 μm) 30 min before and after the induction of LTP along with TPEN (5 μm) and then emetine alone for the entire recording period similar to that of earlier reports (Serrano *et al*., 2005; Sharma *et al*., 2015) resulted in a transient form of LTP. Statistically significant potentiation was observed up to 145 min after STET (119.95 ± 7.2%, Wilcoxon's test, *P *=* *0.042; n = 12) after which the potentials gradually decayed to baseline values (Wilcoxon's test, *P *>* *0.05). STET in presence of TPEN and the NMDA receptor antagonist AP5 (50 μm) completely suppressed the induction of LTP (Fig. 5B; *n* = 8). The fEPSP slope values were not statistically significant at any point compared with the baseline values (Wilcoxon's test, *P *>* *0.05). Thus, the augmented L‐LTP observed in the aged hippocampal neurons after Zn^2+^ chelation was protein synthesis‐ and NMDA receptor dependent, representing a conventional form of LTP that generally takes part in STC (Sajikumar & Frey, 2004).

It has been reported earlier that heterosynaptic co‐activation of NMDA receptors and dopaminergic receptors is necessary for the establishment of L‐LTP and is also same for DA‐LTP (Redondo & Morris, 2011). We tested whether induction and maintenance of long‐lasting DA‐LTP observed in Zn^2+^‐chelated slices of aged rats required NMDAR activity and protein synthesis for its persistence. As shown in Fig. 5C (*n* = 6), co‐application of the protein synthesis inhibitor emetine (20 μm) with TPEN (5 μm) blocked the induction of DA‐LTP. Similarly, co‐application of NMDAR antagonist AP5 (50 μm) and TPEN also completely suppressed the DA‐LTP (Fig. 5D; *n* = 9). The potentiation in both the cases was not significantly different from the baseline values throughout the recording period (Wilcoxon's test, *P *>* *0.05).

### Hippocampal Zn^2+^ chelation re‐establishes synaptic tagging and capture of HFS‐induced LTP and DA‐LTP in aged CA1 pyramidal neurons

**Figure 6:**
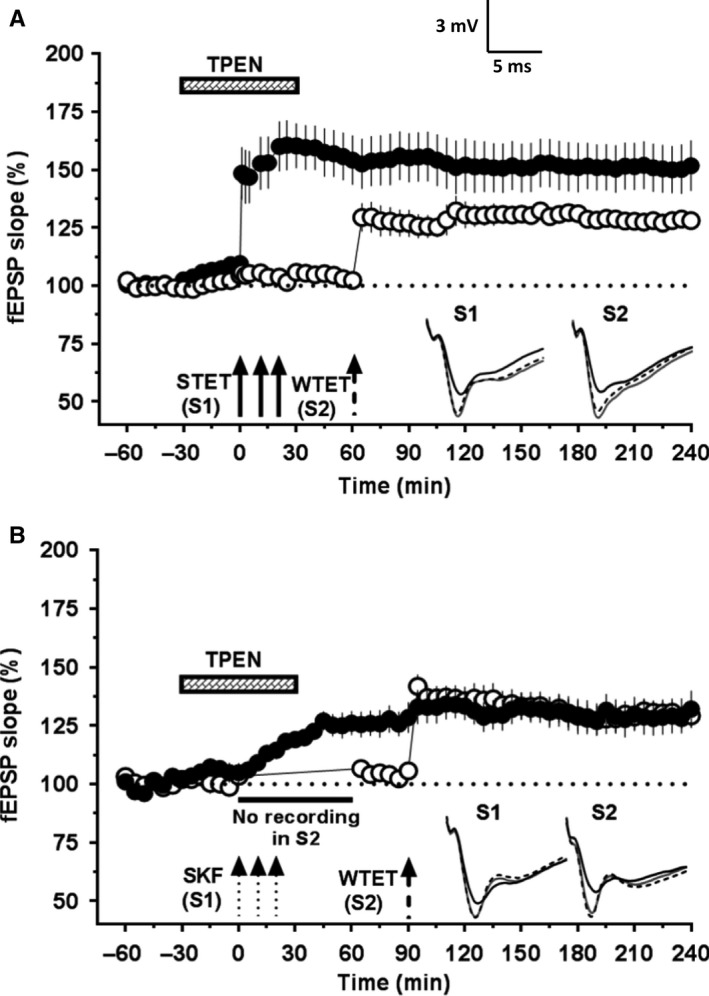
Hippocampal Zn^2+^ chelation re‐establishes synaptic tagging and capture of HFS‐induced L‐LTP and DA‐LTP in aged rats. The location of electrodes for both (A) and (B) was same as in Fig. 2A. (A) The experimental plan is similar as in Fig. 2C, but the slices were also treated with the Zn^2+^ chelator TPEN (5 μm) for 1 h after the baseline recording. STET in the presence of TPEN resulted in significant potentiation in S1 (filled circles) lasting 4 h (Wilcoxon's test, *P *=* *0.004) and also transformed the E‐LTP induced in S2 (open circles) into a long‐lasting one (Wilcoxon's test, *P *=* *0.008; *n* = 9). (B) The experimental plan is similar as in Fig. 2E, but the slices were also treated with TPEN (5 μm) for 1 h after the baseline recording. Application of SKF to aged rat hippocampal slices in the presence of TPEN resulted in restoration of potentiation in S1 (filled circles) that lasted for 4 h (Wilcoxon's test, *P *=* *0.008) and re‐established STC leading to reinforcement of the E‐LTP in S2 (open circles, Wilcoxon's test, *P *=* *0.008, *n* = 8). Insets show representative fEPSP traces for each input recorded at baseline (black solid line), 30 min after the respective tetanization/stimulation in each input (dotted line), and at 240 min (gray solid line). Symbols as in Fig. 1. Scale bar for the traces 3 mV per 5 ms.

As Zn^2+^ chelation in aged hippocampal slices leads to augmentation of L‐LTP magnitude in a protein synthesis‐ and NMDA receptor‐dependent manner, we asked whether the deficits in late‐associative properties such as STC could also have been restored. To test this hypothesis, after a 30‐min stable baseline recording in S1 and S2, TPEN (5 μm) was bath‐applied for the next 60 min (Fig. 6A; *n* = 9). Once 30 min elapsed from the start of TPEN application, STET was delivered to S1 (Fig. 6A; filled circles) for inducing L‐LTP and at the 60th min WTET was delivered to S2 (Fig. 6A; open circles) for inducing E‐LTP. STET‐induced potentiation in S1 was statistically significant immediately after tetanization (+1 min; 148.4 ± 11.2%, Wilcoxon's test *P *=* *0.004) and remained over 4 h (151.7 ± 10.8%, Wilcoxon's test, *P *=* *0.004). The potentiation in S2, which otherwise decayed rapidly, was transformed to restored LTP, expressing STC. Statistically significant potentiation was observed in S2 immediately after WTET (129.4 ± 6.0%, Wilcoxon's test, *P *=* *0.008) until the end of the recording period (128.1 ± 3.2%, Wilcoxon's test, *P *=* *0.008).

Our data suggested that Zn^2+^ chelation restores the deficits in LTP induced by HFS and by dopamine D1/D5 receptor agonists in the hippocampal slices of aged rats. So, we were interested to know whether the Zn^2+^‐chelation can enable the DA‐LTP to take part in STC. If this is the case, an independent nearby synaptic input should be able to benefit from the availability of plasticity factors produced by DA‐LTP. Indeed, we have reported such associative interactions recently in adult rats (Shivarama Shetty *et al*., 2016). We tested the possibility of restored late‐associative properties for DA‐LTP observed upon Zn^2+^ chelation (Fig. 6B; *n* = 8). Here, not only did input S1 (filled circles) show statistically significant potentiation from 10 min onwards (109.13 ± 2.2%; Wilcoxon's test; *P *=* *0.023) and remain over 4 h (132.1 ± 7.7%; Wilcoxon's test; *P *=* *0.008) but also the transient LTP in S2 (open circles) was transformed to L‐LTP, thus showing STC. The mean potentiation in S2 was statistically significant following WTET (141.8 ± 4.9%; Wilcoxon's test; *P *=* *0.008) and remained over 4 h (129.1 ± 5.1%; Wilcoxon's test; *P *=* *0.008). A control series of experiments was conducted wherein prior TPEN application itself did not reinforce the WTET‐induced E‐LTP (Fig. S2).

## Discussion

Aging is associated with synaptic alterations in the hippocampus, a temporal lobe structure critical for declarative memory and cognitive functions (Ojo *et al*., 2015). Aged animals show deficits in LTP induction and the changes in hippocampal plasticity are one of the critical aspects of age‐associated memory impairment in rodents (Burke & Barnes, 2010; Ojo *et al*., 2015). In the present study, we investigated the possible role of Zn^2+^ chelation with respect to aging in re‐establishing synaptic plasticity and associativity in CA1 pyramidal neurons. CA1 neurons have been shown to be preferentially vulnerable to an increase in cytosolic Zn^2+^ that might lead to memory deficits (Takeda & Tamano, 2014).

Our findings demonstrate that the induction and persistence of synaptic plasticity such as tetani‐induced L‐LTP are not completely compromised in aged hippocampal CA3–CA1 synapses but exhibit significant deficit in terms of the magnitude and other functional properties. These results are in line with earlier reports that normal plasticity can be established in aging (Barnes *et al*., 1996; Kumar *et al*., 2007; Sharma *et al*., 2015). Chelation of hippocampal zinc using the same concentration of the chelator in slices of young adult rats showed no significant effects on the induction or maintenance of both tetani‐induced and D1/D5 receptor agonist‐induced LTP. Although some earlier reports have observed an effect of zinc chelation in adult rodent hippocampus on LTP, these reports have mostly focused on the perforant path or mossy fiber synapses (Lu *et al*., 2000; Tamano *et al*., 2015).

It has been suggested earlier that a shift in the mechanisms that regulate the induction of synaptic plasticity rather than a loss of expression mechanisms could be associated with aging (Kumar & Foster, 2007). It can be predicted that the shift in the plasticity mechanisms could restrict the expression of plasticity leading to reduced magnitude of LTP observed in our studies. Indeed, our synaptic tagging and capture experiments support these assumptions as no tagging and capture could be observed in aged hippocampal slices. Successful tagging and capture in healthy neural systems is established by a number of factors such as (i) production and distribution of plasticity factors, (ii) successful setting of synaptic tag that could capture the PRPs, and (iii) requirements of neuromodulatory inputs such as the activation of the dopaminergic fibers (Redondo & Morris, 2011). Our data provide indications for the lack of some of these factors with aging. Lack of STC with aging could be due to the inability of the synaptic populations to synthesize and/or distribute PRPs. We and others have proposed earlier that STC can be successfully established in adult hippocampal networks by the activation of different PRPs, some of them are calcium‐/calmodulin‐dependent protein kinase II (CaMKII), brain‐derived neurotrophic factor (BDNF) and atypical protein kinase C isoform PKMzeta (PKMζ) (Redondo & Morris, 2011; Sajikumar & Korte, 2011). It can be noted that, even in the absence of STC, the L‐LTP maintenance was intact and this finding supports our earlier observation in adult rats that L‐LTP can be established in synaptic populations by PRPs such as BDNF, but STC interactions require specific PRPs such as PKMζ (Sajikumar & Korte, 2011).

Compelling evidences suggest that the activation of neuromodulatory systems, such as the dopaminergic system, influences synaptic plasticity and formation of memory (Hansen & Manahan‐Vaughan, 2014). It has been reported recently in humans that the lack of dopaminergic modulation with aging leads to impairment in associative memory (Chowdhury *et al*., 2012). The lack of dopaminergic receptor‐mediated LTP and STC in our studies strengthens these findings by providing cellular evidence for the inability of aged hippocampal synapses to form and maintain associative plasticity.

What could be the possible reason for the deficit in plasticity and lack of associativity in aged neural networks? Some evidences suggest that cognitive decline may occur due to excess synaptic Zn^2+^ signaling in the hippocampus (Flinn *et al*., 2005; Takeda & Tamano, 2014) and in addition it has been proposed that extracellular zinc levels may rise with age possibly leading to age‐related memory loss and neurodegenerative diseases (Sensi *et al*., 2011). Interestingly, dopaminergic deficit might contribute to the increased zinc levels, similar to the observation that denervation of cholinergic fibers in rat hippocampus leads to increased mossy fiber chelatable zinc levels (Stewart *et al*., 1984).

In our study, we have used a high‐affinity fluorescent Zn^2+^ sensor, FluoZin‐3, to visualize Zn^2+^ levels in the hippocampal slices and the results reveal significantly high levels of zinc in the hippocampal regions of the aged rats. Given that the probe we have used is cell permeable (FluoZin‐3, AM), the signal is expected to be mostly arising from the intracellular pool (Gee *et al*., 2002). Also, because of the technical limitations, we have used the slices after fixation with 4% PFA, which might affect the esterase cleavage of the acetoxymethyl ester (AM) probe, and as a result may not trap the probe inside the cells. This, however, would not affect the probe entry into the intracellular compartment. Therefore, we have followed a single brief rinse to remove the unbound probe and then imaging immediately. Given that the imaging conditions followed were same for both the adult and aged rat slices, the observed difference in FluoZin‐3 signal seems to indicate increased intracellular zinc levels in the hippocampus of aged rats. Nonetheless, zinc imaging with fluorescent probes has limitations in specificity, selectivity, and sensitivity (detection limit) depending on the property of the probes/sensors and on the experimental conditions (Hawkins *et al*., 2012; Maret, 2015). Further, more sophisticated methods for estimation of zinc levels in the hippocampus would help to confirm these findings.

Accumulation of zinc with aging and its possible role in interfering with plasticity and associativity is so far not addressed and we provide the first evidence that administration of zinc chelators has a positive effect in restoring plasticity and associativity. With advanced aging, zinc transporter function and/or levels may be altered (Saito *et al*., 2000; Adlard *et al*., 2010) that can lead to the accumulation of chelatable zinc in neurons. Although zinc nutritional deficiency is reported in advanced aging, studies in rodents provided mechanistic evidence that systemic zinc deficiency increases brain zinc retention by suppressing the zinc transporter ZnT1 levels (Takeda *et al*., 2001). Another possible reason for the accumulation of zinc in aging could be due to biological stress, which is suggested to induce excess intracellular zinc signaling in the hippocampus, followed by hippocampus‐dependent memory deficits (Takeda & Tamano, 2014).

Neurons have relatively large stores of Zn^2+^ associated with proteins such as metallothionein‐III (MTIII) and this can be readily released following excitotoxic and oxidative insults (Vander Jagt *et al*., 2009). Intracellular organelles also have significant stores of Zn^2+^ (Sensi *et al*., 2009). A number of factors could contribute to the increased Zn^2+^ levels in aging: (i) inhibition of Zn^2+^ export by products of lipid peroxidation, such as 4‐hydroxynonenal (Smith *et al*., 2006), (ii) systemic Zn^2+^ deficiency leading to increased brain Zn^2+^ retention by suppressing ZnT1 levels (Takeda *et al*., 2001; Chowanadisai *et al*., 2005), (iii) intracellular acidosis leading to enhanced Zn^2+^ influx through the activation of the H^+^–Zn^2+^ exchanger (Sensi *et al*., 2003; Frazzini *et al*., 2007), and (iv) ischemia‐driven [Zn^2+^]_i_ rises as the result of a combined process of Zn^2+^ influx and Zn^2+^ release from intracellular stores (Sensi *et al*., 2009).

The deficits in dopaminergic signaling and the associativity impairments could be related to alterations in the NMDA receptor functions as its functions are also regulated by zinc (Paoletti *et al*., 1997; Rachline *et al*., 2005; Izumi *et al*., 2006; Zhu *et al*., 2012). Chelating zinc might have fine‐tuned NMDA receptor functions to a degree that permits the establishment of long‐term plasticity and STC. NMDA receptor‐dependent plasticity is critical for the establishment of STC in adult CA3–CA1 synapses (Redondo & Morris, 2011), although we do not rule out the possibility of impairments in multiple mechanisms associated with excessive zinc that leads to plasticity deficits. Zinc also inhibits biological activities of BDNF and other neurotrophins (Ross *et al*., 1997), and BDNF is an important plasticity‐related protein necessary for the maintenance of L‐LTP and STC (Lu *et al*., 2011).

The strategy of using weak zinc chelators and pro‐chelators has proven promising in AD animal models and in human studies with AD patients (Ritchie *et al*., 2003; Lee *et al*., 2004). Our study provides interesting hints regarding synaptic plasticity impairments in aged hippocampal neurons resulting from zinc accumulation and the possible beneficial effects of its regulation by pharmacological chelating agents. Our findings have important implications in understanding the adverse consequences of zinc accumulation on synaptic plasticity mechanisms, and learning and memory functions.

## Experimental procedures

### Electrophysiology

A total of 143 acute hippocampal slices from twenty‐seven 82‐ to 84‐week‐old male Wistar rats and 44 slices from twenty‐one 5‐ to 7‐week‐old male Wistar rats were used for electrophysiological recordings. Animals were maintained on a 12‐h/12‐h light/dark cycle, with food and water available *ad libitum*. All animal procedures were carried out in accordance with protocols R13‐4656(A)13 and R13‐5711(A1)14 approved by the Institutional Animal Care and Use Committees (IACUC) at the National University of Singapore. Briefly, after anesthetization using CO_2_, the rats were decapitated and the brains were quickly removed and transferred into cold (2–4 °C), oxygenated ACSF. The ACSF contained the following (in millimolars): 124 NaCl, 3.7 KCl, 1.0 MgSO_4_·7H_2_O, 2.5 CaCl_2_·2H_2_O, 1.2 KH_2_PO_4_, 24.6 NaHCO_3_, and 10 D‐glucose, equilibrated with 95% O_2_–5% CO_2_ (carbogen; total consumption 16 L h^−1^). From the right hippocampus of each rat, 8–10 transverse hippocampal slices (400 μm thick) were prepared using a manual tissue slicer. The slices were incubated in an interface brain slice chamber (Scientific Systems Design, Mississauga, Ontario, Canada) at 32 °C for three hours at an ACSF flow rate of 1 mL min^−1^. As the number of aged animals was limited, the slices from each rat were utilized maximally by simultaneously performing different experiments in four interphase chambers.

In one‐pathway experiments, one monopolar stainless steel electrode (5 MΩ; A‐M Systems, Sequim, WA, USA) was positioned within the stratum radiatum of the CA1 region for stimulating the synaptic input‐1 (S1; Fig. 1A). In two‐pathway synaptic tagging and capture experiments, two electrodes were positioned within the stratum radiatum of the CA1 region for stimulating two independent synaptic inputs S1 and S2, to a common neuronal population (Fig. 2A). Pathway independence between S1 and S2 was tested using standard paired‐pulse stimulation protocol described previously (Sajikumar & Korte, 2011). In all experiments, one monopolar stainless steel electrode (5 MΩ; A‐M Systems, Sequim, WA, USA) was placed in the CA1 apical dendritic layer for recording the fEPSP (measured as its slope function). Signals were amplified by a differential amplifier (Model 1700; AM Systems) and digitized using a CED 1401 analog‐to‐digital converter (Cambridge Electronic Design, Cambridge, UK) and monitored online.

Input–output relation (afferent stimulation vs. fEPSP slope) was determined for each slice and the stimulus intensity that evokes 40% of the maximum fEPSP slope is set as test stimulus intensity. For baseline recording and testing at each time point, four 0.2‐Hz biphasic constant‐current pulses (0.1 ms per polarity) were used. L‐LTP was induced using three stimulus trains of 100 pulses (‘strong’ tetanus [STET], 100 Hz; duration, 0.2 ms per polarity; intertrain interval, 10 min). E‐LTP was induced with a WTET protocol consisting of one 100‐Hz train (WTET; 21 biphasic constant‐current pulses; pulse duration per half‐wave 0.2 ms) (Shetty *et al*., 2015). In all experiments, a stable baseline was recorded for at least 30 min.

#### Drugs

The D1/D5 receptor agonist SKF‐38393 hydrochloride [(±)‐1‐Phenyl‐2,3,4,5‐ tetrahydro‐(1H)‐3‐benzazepine‐7,8‐diol hydrochloride] (SKF; #D047; Sigma‐Aldrich, Singapore) was stored at −20 °C as a 50‐mm stock in deionised water. The stocks were used within a week. The Zn^2+^ chelator TPEN [*N*,*N*,*N*′,*N*′‐Tetrakis (2‐pyridylmethyl) ethylenediamine] (Tocris Bioscience, Bristol, UK) was stored as a 25‐mm stock in dimethyl sulfoxide (DMSO) at −20 °C. Emetine dihydrochloride hydrate (Sigma‐Aldrich, Singapore) and d‐2‐amino‐5‐phosphonopentanoic acid (AP‐5) (Tocris Bioscience, Bristol, UK) were prepared as concentrated stock solutions in DMSO and were diluted in ACSF to obtain a final concentration of 20 and 50 μm, respectively. Light‐sensitive drugs were protected from light during storage and bath application. Prior to application, the drug stocks were diluted to the final concentration in ACSF, equilibrated with carbogen, and bath‐applied for specified durations. Whenever the stocks are prepared in DMSO, the final DMSO concentration was kept below 0.1%, a concentration which has been shown to not affect basal synaptic responses (Navakkode *et al*., 2005).

#### **Zn^2+^ imaging with FluoZin^TM^‐3, AM**

Cell‐permeable, Zn^2+^‐selective fluorescent indicator FluoZin^TM^‐3 (AM) was purchased from ThermoFisher Scientific, Singapore (Molecular Probes, #F24195), stored at −20 °C as aliquots of 2‐mm stock in DMSO, and diluted in ACSF to working concentration of 2 μm immediately prior to use. Acute hippocampal slices of 400 μm thickness were prepared from the left hippocampus of adult and aged rats as described above. The slices were then incubated in a submerged holding chamber for 2 h at 32 °C in ACSF. After the incubation period, the slices were carefully collected and stored in 4% PFA in phosphate‐buffered saline (PBS; pH 7.4) at 4 °C. On the day of imaging, the slices were rinsed three times in ACSF and treated with 2 μm FluoZin^TM^‐3 solution for ten minutes in dark. Then the slices were rinsed once with ACSF to wash off the unbound FluoZin^TM^‐3, carefully transferred to a glass‐bottomed imaging dish, and imaged using Zeiss LSM 710 confocal microscope.

#### Image acquisition

The images were acquired with an inverted Zeiss Axiovert LSM 710 confocal microscope (Carl Zeiss, Oberkochen, Germany) using Zen Black software. The excitation source was 543 nm He–Ne laser. The objective lens used was 10× (numerical aperture 0.4). Images were acquired from the middle layer of the slice, and each image was stitched with Tile Scan mode (4 × 3 frames). All the settings and acquisition parameters were kept constant for all the images.

#### Image analysis

The acquired raw images were analyzed using the ImageJ 1.50i software (NIH, Bethesda, Maryland). For the quantification of the fluorescence intensity from the whole slice, entire field of view containing a single slice was used (all the images had the same image properties). The background signal was negligible in all the images. Auto‐fluorescence from the unstained slice was checked and was almost nil (data not shown). The raw intensity density of the FluoZin‐3 fluorescence from the whole slice was measured from the adult and aged rat slices. The raw intensity density of the two groups was expressed as mean ± SEM.

Further, the raw intensity density of the FluoZin‐3 fluorescence from the CA1 region was measured. The CA1 region was selected as the region of interest (ROI) in each image by drawing a polygon along the CA2–CA1 border, the alveolar border of CA1, and CA1–subicular border and along the hippocampal fissure between the DG molecular layer and the lacunosum molecular layer of CA1. The raw intensity density from the ROI in each slice was normalized to the area of ROI. The raw intensity density of the two groups was expressed as mean ± SEM. The data were subjected to statistical analysis using GraphPad Prism 6.0 (La Jolla, California). In both cases, unpaired t‐test with Welch's correction was used for comparison.

### Statistical analysis

The time‐matched, normalized data were averaged from replicate experiments and represented as mean ± SEM. The fEPSP slope values expressed as percentages of average baseline values per time point were subjected to statistical analysis using GraphPad Prism 6.0. Considering the normality violations at small sample numbers, nonparametric tests were used. Wilcoxon's matched‐pairs signed rank test was used to analyze the average values of the slope function of the fEPSP (mV ms^−1^) per time point when compared within the group and Mann–Whitney *U*‐test was used for between‐group comparisons. Statistical significance was assumed at *P *<* *0.05.

## Author contributions

S.S. designed research; M.S. and M.S.S. performed research; and M.S., M.S.S., and S.S. analyzed data and wrote the paper.

## Conflict of interest

None declared.

## Funding

S.S is supported by National Medical Research Council Collaborative Research Grant (NMRC‐CBRG‐0041/2013 and NMRC/CBRG/0099/2015), Ministry of Education Academic Research Funding (MOE AcRF‐ Tier 1 – T1‐2012 Oct ‐02) and NUS‐Strategic and Aspiration Research Funds (for Memory Networks Programme). M.S. and M.S.S. are supported by NUS Research Scholarship.

## Supporting information


**Fig. S1** Effect of zinc chelation on basal synaptic responses.

**Fig. S2** Effect of prior zinc chelation on E‐LTP in aged slices.
