# Supplementary figures and images for: Chelation of hippocampal zinc enhances long‐term potentiation and synaptic tagging/capture in CA1 pyramidal neurons of aged rats: implications to aging and memory
Source: Aging Cell. 2016 Sep 16;16(1):136–48. doi: 10.1111/acel.12537 (PMC5242293; doi:10.1111/acel.12537)

**A**

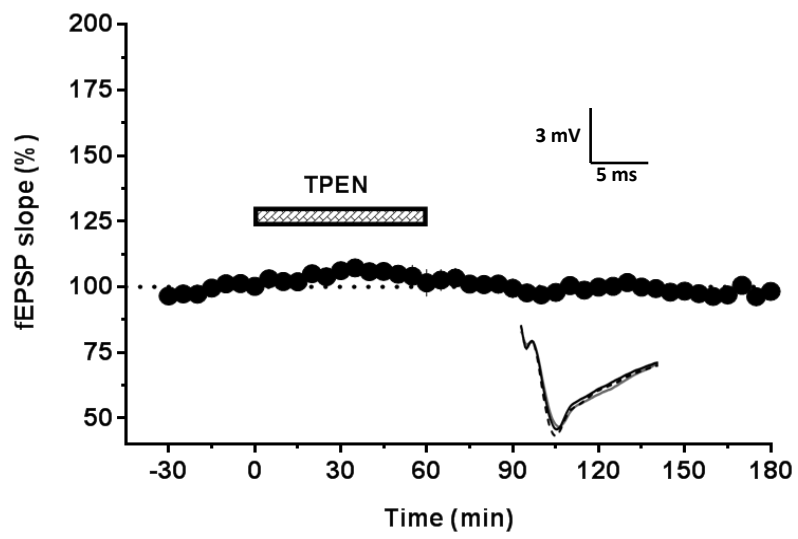

*Shetty et al., 2016*  
*Supplementary Figure 1*

Supplement: Supplementary file 1 — Fig. S1 Effect of zinc chelation on basal synaptic responses. [file ACEL-16-136-s001.pdf]

**A**

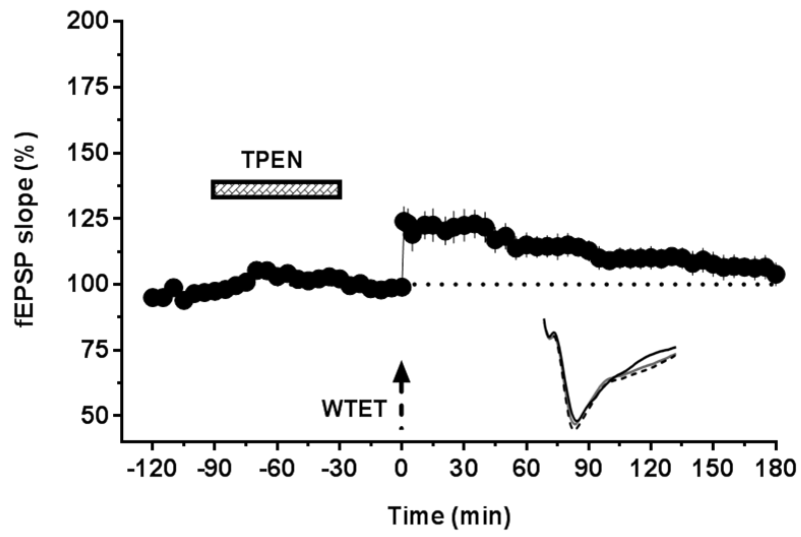

*Shetty et al., 2016*  
*Supplementary Figure 2*

Supplement: Supplementary file 2 — Fig. S2 Effect of prior zinc chelation on E‐LTP in aged slices. [file ACEL-16-136-s002.pdf]
